# Vacuolar Protein Sorting Genes in Parkinson's Disease: A Re-appraisal of Mutations Detection Rate and Neurobiology of Disease

**DOI:** 10.3389/fnins.2016.00532

**Published:** 2016-11-24

**Authors:** Stefano Gambardella, Francesca Biagioni, Rosangela Ferese, Carla L. Busceti, Alessandro Frati, Giuseppe Novelli, Stefano Ruggieri, Francesco Fornai

**Affiliations:** ^1^IRCCS NeuromedPozzilli, Italy; ^2^Department of Biomedicine and Prevention, School of Medicine, University of Rome ‘Tor Vergata’Rome, Italy; ^3^Department of Translational Research and New Technologies in Medicine and Surgery, University of PisaPisa, Italy

**Keywords:** retromers, VPS, protein clearance, autophagoproteasome, neurogenetics, genetic parkinsonism

## Abstract

Mammalian retromers play a critical role in protein trans-membrane sorting from endosome to the trans-Golgi network (TGN). Recently, retromer alterations have been related to the onset of Parkinson's Disease (PD) since the variant p.Asp620Asn in VPS35 (Vacuolar Protein Sorting 35) was identified as a cause of late onset PD. This variant causes a primary defect in endosomal trafficking and retromers formation. Other mutations in VPS genes have been reported in both sporadic and familial PD. These mutations are less defined. Understanding the specific prevalence of all VPS gene mutations is key to understand the relevance of retromers impairment in the onset of PD. A number of PD-related mutations despite affecting different biochemical systems (autophagy, mitophagy, proteasome, endosomes, protein folding), all converge in producing an impairment in cell clearance. This may explain how genetic predispositions to PD may derive from slightly deleterious VPS mutations when combined with environmental agents overwhelming the clearance of the cell. This manuscript reviews genetic data produced in the last 5 years to re-define the actual prevalence of VPS gene mutations in the onset of PD. The prevalence of p.Asp620Asn mutation in VPS35 is 0.286 of familial PD. This increases up to 0.548 when considering mutations affecting all VPS genes. This configures mutations in VPS genes as the second most frequent autosomal dominant PD genotype. This high prevalence, joined with increased awareness of the role played by retromers in the neurobiology of PD, suggests environmentally-induced VPS alterations as crucial in the genesis of PD.

## Introduction

Mammalian retromer is a protein complex composed of three vacuolar protein sorting (VPS) 26, 29, and 35, with an important role in trafficking trans-membrane receptors toward the endosome compartment (Nothwehr et al., 1999; Seaman, 2012).

In 2011, two different studies carried out in selected kindreds affected by Parkinson's disease (PD) by using next-generation sequencing (NGS) identified an aspartic-acid-to-asparagine mutation within VPS35 gene (p.Asp620Asn) (Vilariño-Güell et al., 2011; Zimprich et al., 2011). *In silico* analysis showed that amino acid Asp620 within VPS35 gene is highly conserved from yeast to humans. Moreover, studies based on molecular dynamics simulations predict that the variant p.Asp620Asn, is a deleterious substitution since it reduces salt bridges, which in turn produce an increase in protein flexibility (Vilariño-Güell et al., 2011; Zimprich et al., 2011).

As reported by Trinh et al. (2014), the cumulative incidence of VPS35 p.Asp620Asn has a lower quartile when considering age at onset <45 years and an upper quartile when considering age at onset >59 years. This mutation has a high but incomplete penetrance. Clinically, unaffected carriers have been reported. Four carriers between 49 and 67 years at the time of exam (Vilariño-Güell et al., 2011), and three carriers younger than 60 years (Zimprich et al., 2011) have been described so far. It still remains to be established whether these mutations are really non-penetrant or these patients were evaluated at a pre-symptomatic stage.

Incomplete penetrance would be consistent with slight consequences produced by this mutation, which minimally affects the association with VPS29 and VPS26 to constitute the whole retromer complex. In fact, this mutation of VPS35 produces a protein with abnormally flexibility, but it remains correctly folded and binds VPS29 and VPS26a with the same affinity of wild-type VPS35 (Follett et al., 2014).

Conversely, in the hypothesis that this mutation possesses almost full penetrance, this may produce severe alterations in endosomal morphology and trafficking. In line with this, p.Asp620Asn causes retromer misplacement toward a perinuclear area, as witnessed by enlarged stagnant perinuclear endosomes described in a PD patient (Follett et al., 2014; Tsika et al., 2014).

In addition, the mutation p.Asp620Asn despite not altering the binding of VPS35 with VPS29 and VPS26a, it does impair the binding of VPS35 with FAM21-containing WASH complex, which mediates the production of branched actin networks on the surface of the endosomal membrane (McGough et al., 2014; Zavodszky et al., 2014; Tsuyoshi and Yuzuru, 2015). This alters the trafficking of cathepsin D, which is responsible for the degradation of a number of proteins including α-synuclein (McGlinchey and Lee, 2015).

However, the molecular mechanisms which lead from this VPS mutation to neurodegeneration remain unclear. Recent studies demonstrate that all VPS35 mutations in PD cause mitochondrial fragmentation and neuronal death (Tsika et al., 2014; Zavodszky et al., 2014). These effects, linking VPS35 to mitochondrial homeostasis, reveal a novel mechanism of disease. This is in line with findings showing that autophagy is impaired in cells expressing VPS35 mutations. The impairment of autophagy and mitochondrial turnover in association with altered kinetics of exosomes may depend on abnormal trafficking of the autophagy protein ATG9A which indeed occurs in this PD genotype (Haelterman et al., 2014).

## Prevalence of VPS35 variants in familial and sporadic PD patients

Several analysis have been carried out in PD patients in order to establish the contribution of VPS35 mutations to the onset of PD. From 2011, 15 case-control studies worldwide analyzed a total 21,824 PD patients. Some of these studies considered familial (F)- and/or sporadic (S)-PD as independent populations, while others considered S-PD and F-PD as mixed population (M-PD). In these latter studies the occurrence/absence of the disease was investigated only in the families of the carrier. Based on these studies, an accurate re-appraisal of literature allows to re-define the prevalence of VPS mutations in M-PD, S-PD, and F-PD (Table 1; Vilariño-Güell et al., 2011; Zimprich et al., 2011; Ando et al., 2012; Deng et al., 2012; Guella et al., 2012; Guo et al., 2012; Kumar et al., 2012; Lesage et al., 2012; Sharma et al., 2012; Sheerin et al., 2012; Zhang et al., 2012; Chen et al., 2013; Sudhaman et al., 2013; Blanckenberg et al., 2014; Gagliardi et al., 2014).

**Table 1:** **Prevalence of mutations in VPS genes**.

**Gene**	**Variant**	**References**	**Patients Analyzed**		
			**M-PD**	**F-PD**	**S-PD**
VPS35	Asp620Asn	Sharma et al., 2012	8870 (7)	nr (5)	nr (2)
		Vilariño-Güell et al., 2011	4432 (5)	nr (4)	nr (1)
		Zimprich et al., 2011	1348 (4)	nr (4)	nr (0)
		Ando et al., 2012	733 (4)	300 (3)	433 (1)
		Deng et al., 2012	202 (0)	72 (0)	130 (0)
		Chen et al., 2013	609 (0)	37 (0)	572 (0)
		Guella et al., 2012	475 (0)	475 (0)	nr
		Guo et al., 2012	1038 (0)	27 (0)	1011 (0)
		Kumar et al., 2012	1774 (1)	539 (1)	1235 (0)
		Sheerin et al., 2012	597 (1)	335 (1)	262 (0)
		Sudhaman et al., 2013	320 (0)	69 (0)	251 (0)
		Lesage et al., 2012	246 (3)	246 (3)	nr
		Blanckenberg et al., 2014	418 (0)	418 (0)	nr
		Zhang et al., 2012	512 (0)	32 (0)	480 (0)
		Gagliardi et al., 2014	250 (0)	250 (0)	nr
		**Tot**	**21,824 (25)**	**2800 (8)**	**4374 (1)**
		**%**	**0.115**	**0.286**	**0.023**
	Leu774Met	Sharma et al., 2012	8870 (6)	nr	nr
		Zimprich et al., 2011	862 (2)	nr	nr
	Pro316Ser	Vilariño-Güell et al., 2011	4515 (1)	nr (1)	nr (0)
	Arg524Trp	Zimprich et al., 2011	862 (1)	nr	nr
	Ile241Met	Zimprich et al., 2011	862 (1)	nr	nr
	Met57Ile	Zimprich et al., 2011	862 (1)	nr	nr
	Gly51Ser	Sharma et al., 2012	8870 (3)	nr	na
		**Tot**	**14,247 (15)**		
		**%**	**0.105**		
VPS26a	Lys93Glu	Shannon et al., 2014	1906 (0)	nr	nr
	Lys93Glu	Gustavsson et al., 2015	396 (1)	nr	nr
	Met112Ile	Koschmidder et al., 2014	245	nr	nr
	Met112Val	Gustavsson et al., 2015	396 (1)	nr	nr
	Lys297X	Gustavsson et al., 2015	396 (1)	nr	nr
	Pro316Ser	Koschmidder et al., 2014	245 (0)	nr	nr
VPS29	Asn72His	Shannon et al., 2014	1906 (1)	nr	nr
		**Tot**	**2547 (4)**		
		**%**	**0.157**		

*Variants of VPS26a, 29 and 35 are shown. For each variant the table reports the published reference, the number of patient analyzed in each publication, the number of mutation detected (shown in brackets) in each publication for Sporadic (S-PD), Familiar (F-PD), and mixed population (M-PD = S-PD + F-PD). Box 1 shows data related to variant Asp620Asn of VPS35; Box 2 shows data related to other variants of VPS35; Box 3 shows data related to variants of VPS26a and VPS29. Each box reports in bold style: (i) the total number of patients analyzed for each population, (ii) the number of variants identified (in bracket), and (iii) the prevalence of each variant expressed as percentage of the population analyzed. Nr, not reported %, percentage*.

### M-PD

Total prevalence of VPS35 mutations in M-PD derive mostly from the mutation p.Asp620Asn and rarely from other mutations. The frequent p.Asp620Asn mutation has been genotyped in a total of 21,824 M-PD, and it was detected in 25 patients (prevalence of 0.115%, Table 1). Among these, only 21 patients were affected by PD at the time of genetic test (Vilariño-Güell et al., 2011; Zimprich et al., 2011; Sharma et al., 2012).

On the other hand, data on rare variants are limited to genetic studies which considered the whole VPS35 gene sequence. The most important variants being detected are p.Pro316Ser (1/4515, M-PD) (Vilariño-Güell et al., 2011), p.Arg524Trp (1/860 S-PD) and p.Ile241Met (1/860, F-PD) (Chen et al., 2013), p.Leu774Met (2/860PD and 6/8870) and p.Gly51Ser (3/8870) (Zimprich et al., 2011; Sharma et al., 2012). Further analyses of these variants are strongly required in order to understand their contribution to the onset of PD. In fact, while p.Arg524Trp and p.Ile241Met are predicted to be deleterious by molecular dynamics analyses, the pathogenic role of p.Pro316Ser remains uncertain. Further studies are required to ascertain the pathogenic effects of the mutation p.Leu774Met (which owns a limited impact on protein stability), while the significance of the mutation p.Gly51Ser remains unclear.

Assuming pathological effects for all these VPS35 rare variants, their prevalence occurs in up to 0.105% (15/14247) of M-PD (Table 1). When adding the prevalence of these rare VPS35 mutations to the most common p.Asp620Asn mutation (0.115%), the overall prevalence of VPS35 variants reaches 0.220% which potentially contributes to M-PD.

### F-PD and S-PD

Data on the specific contribution of VPS35 variants in F-PD and S-PD can be obtained from case-control studies which consider F-PD or S-PD as distinct populations (Ando et al., 2012; Deng et al., 2012; Guella et al., 2012; Guo et al., 2012; Kumar et al., 2012; Lesage et al., 2012; Sheerin et al., 2012; Zhang et al., 2012; Chen et al., 2013; Sudhaman et al., 2013; Blanckenberg et al., 2014; Gagliardi et al., 2014). These studies analyzed a total of 7174 PD patients, 2800 with F-PD and 4374 with S-PD. Data were obtained only for the most common mutation p.Asp620Asn, which was detected in 1 sporadic (1/4374, 0.023%) and 8 familial (8/2800, 0.286%) patients (Table 1). When considering the prevalence of F-PD compared with M-PD, the prevalence of p.Asp620Asn calculated in a pure F-PD population increases up to 10-fold compared with M-PD (two-tailed *P* = 0.0032).

Assuming the prevalence of rare VPS mutations from M-PD studies (0.105%) as due to F-PD, the prevalence of all VPS35 variants in F-PD (both p.Asp620Asn and all rare variants) is 0.391% (0.286% of p.Asp620Asn in F-PD + 0.105 % or rare variants in M-PD).

This is in line with what reported by Deng et al. (2013), who considered VPS35 as the second most frequent cause of late-onset F-PD (age at onset > 50 years), after LRRK2 mutations.

## Variants in the cargo binding trimeric subcomplex of the retromer

Given the pathogenic effects of VPS35 mutations, other studies aimed to establish the potential contribution to PD of other VPS genes encoding for trimeric sub-complex of retromer. Genetic screening on the whole VPS26a, VPS26b, and VPS29 genes have identified other VPS mutations which may produce pathogenic effects both in PD and atypical parkinsonisms, such as PSP (Progressive Supranuclear Palsy), MSA (Multiple system atrophy), and Lewy body dementia (LBD) (Koschmidder et al., 2014; Shannon et al., 2014; Gustavsson et al., 2015). These studies considered S-PD and F-PD as M-PD, and they reported the occurrence/absence of the disease only in the families of the carriers.

When considering only those variants which are very likely to produce deleterious effect, five mutations need to be considered in VPS26a, one in VPS29, while no potentially pathogenic variants are described so far in VPS26b. In depth analysis is required to rule out definitely the pathogenic role of VPS26b variants. In fact, such a lack of deleterious effects would be unexpected when considering that VPS26b compete with VPS26A for a single-binding site on VPS35 (Bugarcic et al., 2011). All variants detected so far in VPS26a are placed outside the VPS35 binding site (amino acids 235–246), being unlikely to affect interactions between subunits of VPS35. For instance p.Lys93Glu (c.A277G) in exon 4 is placed in a highly conserved region of the VPS26a N-domain (amino acids 6-148), which binds the receptor and promotes its internalization (Shi et al., 2006). Although no functional studies have been reported, this mutation may affect the binding to protein cargoes (Gustavsson et al., 2015). This mutation has been identified in a Canadian female (age at onset = 58) affected by MSA, and it was detected in her sister who was also affected by MSA (Gustavsson et al., 2015). Another case-control analysis identified this variant in a proband with F-PD (age at onset = 56) and in two non-affected siblings of her, suggesting an incomplete penetrance or an uncertain pathogenic effect (Shannon et al., 2014). Therefore, p.Lys93Glu has been identified in sporadic and atypical PD patients.

Variation of codon 112 has been reported in two independent analyses. A mutation p.Met112Ile, caused by a substitution in the last nucleotide of the codon (c.336G>C) was reported in a S-PD (Koschmidder et al., 2014), while p.Met112Val (c.334 A>G) was identified in a patient with atypical PD (Gustavsson et al., 2015). Similarly to aminoacid Lys93, Met112 takes place on an external protein domain, and it may impair the interaction of VPS26a with other retromer proteins (Gustavsson et al., 2015). Interestingly, this variant was predicted to be benign by four of the five *in silico* prediction software being tested, and its effects remain to be evaluated in other PD patients (Koschmidder et al., 2014; Gustavsson et al., 2015).

Other variants are placed in exon 9. For instance, p.Lys297X has been identified in a Chamorro patient from Guam with sporadic PSP (Gustavsson et al., 2015), while p.Pro316Ser has been detected in a patient with S-PD, but even this mutation was predicted to be a benign variant by *in silico* analysis. The effects of exon 9 mutations on retromer functions are currently unknown (Koschmidder et al., 2014).

In the VPS29 gene, variant p.Asn72His was identified in one proband with PD (age at onset = 70). Even this variant, predicted to be neutral, is placed outside the VPS35 binding site of VPS29 and it is unlikely to affect its binding to VPS35 (Shannon et al., 2014).

Taken together, all VPS studies (except VPS35) identified 4 variants in M-PD (4/2547, 0.157%, Table 1), and 3 variants in atypical PD (3/229, 1.31%), confirming the relevance of genetic analysis of the trimeric sub-complex of the retromer both in PD and atypical parkinsonism.

Cumulative prevalence of variants in all VPS genes in M-PD is 0.377% (0.220% of all VPS35 variants in M-PD+ 0.157% of all VPS26a, VPS26b, VPS29 variants in M-PD).

When considering the prevalence of mutations in VPS26a, VPS26b, and VPS29 (0.157%) as calculated only from mixed population (M-PD) studies, this corresponds to 0.157%. When arbitrarily including these mutations as part of F-PD, the prevalence of all VPS genes variants in F-PD rises up to 0.548% (0.391% of all VPS35 variants in F-PD + 0.157 % of VPS26a, VPS26b, and VPS29 variants in M-PD; Table 1).

## VPS and neurobiology of PD

**Figure 1:**
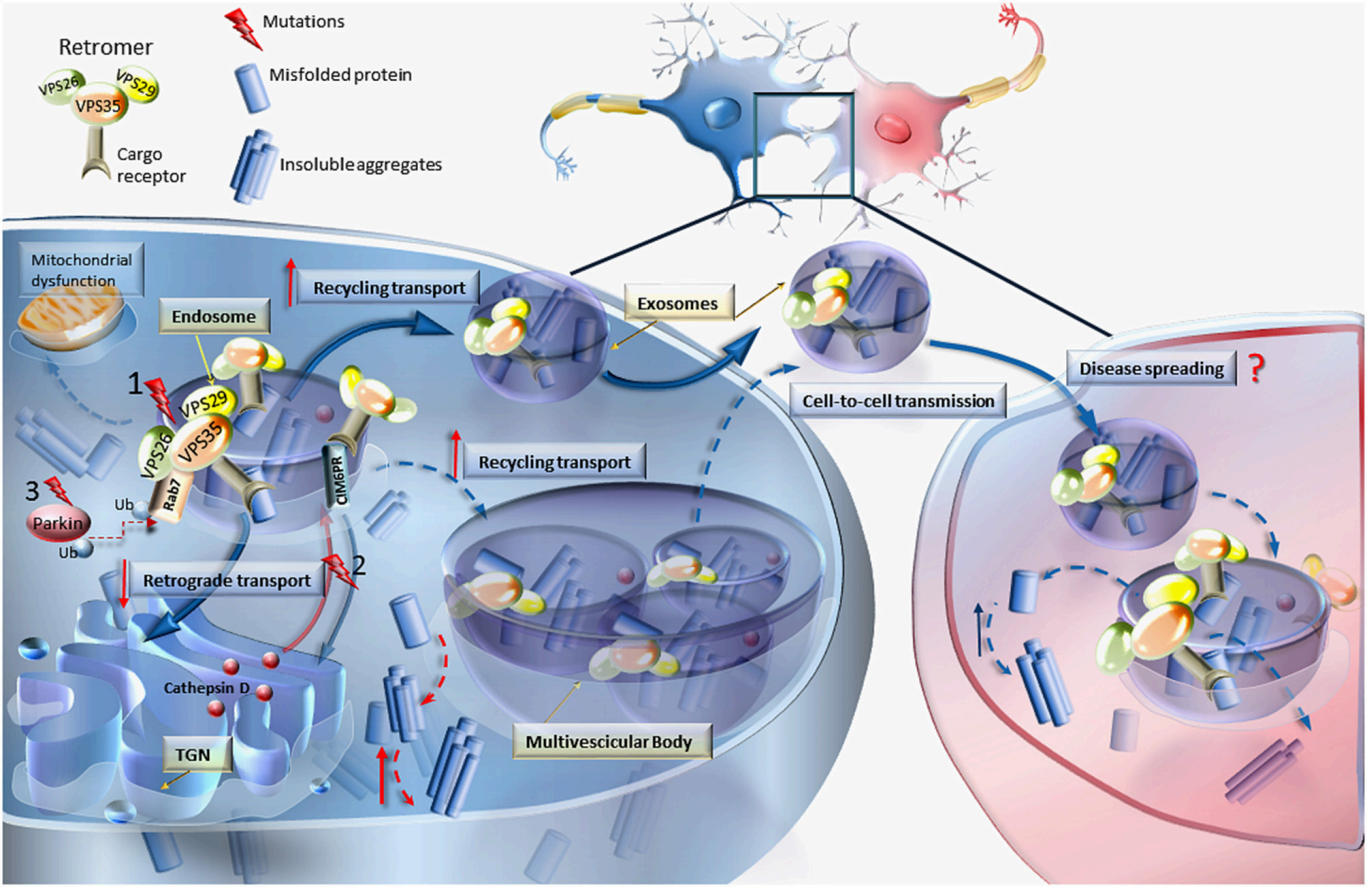
**VPS mutations and retromer dysfunction**. This cartoon reports the most relevant effects of VPS dysfunction on the molecular mechanisms involved in cellular trafficking. Mutations (nr.1) occurring in any of the Vacuolar Protein Sorting components of the retromer (VPS35, VPS26, and VPS29) may lead to increased levels of misfolded proteins, thereby causing abnormal sorting and trafficking. In an attempt to get rid of misfolded proteins, the routine trafficking may shift from endosomes and trans-Golgi network (TGN) to the cell membrane to produce the release of aberrant cargoes. Diffusible retromers may be key in neurodegenerative disorders, posing this unconventional mechanism of cell-to-cell communication as a cause of disease spreading. Retromer dysfunction may also derive from mutations (nr.2) impairing the retrograde transport of cation-independent mannose-6-phosphate receptor (CIM6PR), which in turn becomes unable to bind cathepsin D and other proteases to the TGN, to be delivered to the endosome (Miura et al., 2014). Since cathepsin D is an endosome–lysosome protease which is crucial for degrading α-synuclein, this may explain the occurrence of α-synuclein accumulation also in the course of retromer-related PD. The relevance of the endosomal/retromer/exosome compartment in PD is supported by the interaction of VPS35 with parkin by promoting Rab7 ubiquitination. In fact, Parkin mutations (nr.3) are associated with retromer dysfunctions (Song et al., 2016). In line with consistent finding on mitochondrial alterations in PD, VPS35 was shown to modulate mitochondrial integrity and mitochondrial turnover (Tang et al., 2015b; Wang et al., 2016).

The re-appraisal of the prevalence of VPS genetic alterations, apart from re-defining the relevance of this class of mutations in causing F-PD, is helpful to analyze the potential dysfunctions of VPS in producing sporadic PD. In fact, the process of disclosing genetic determinants of PD provides molecular markers which often are shared by all (genetic and sporadic) PD cases. The proof of principle is represented by the case of alpha synuclein, which is responsible for rare F-PD but it is found to be altered at molecular level in almost all PD phenotypes where it represents the hallmark of proteinaceous aggregates known as Lewy Bodies (Spillantini et al., 1997). In fact, alpha synuclein provided the basis to unravel the general mechanisms of action of misfolded proteins, which is relevant to interpret the disease course both in familial and sporadic PD (Fornai et al., 2005a,b,c, 2006, 2008; Giorgi et al., 2006; Iacovelli et al., 2006; Mauceli et al., 2006; Lazzeri et al., 2007; Ferrucci et al., 2008). In keeping with this, the role of VPS is key in releasing protein-enriched exosomes. This is critical to understand disease propagation through synaptically connected regions along the whole CNS. In this way, VPS mutations may be a pivot to understand cell-to-cell transmission of protein cargoes through exosomes (Danzer et al., 2012; Poehler et al., 2014; Tsunemi et al., 2014; Emmanouilidou and Vekrellis, 2016; Lööv et al., 2016; Figure 1). Thus, an in depth analysis of VPS alterations is expected to disclose the anatomical basis of disease progression as recently described (Hawkes et al., 2010; Del Tredici and Braak, 2013; Holmqvist et al., 2014; Garcia-Esparcia et al., 2015; Lamberts et al., 2015). Within this scenario one should consider that VPS is involved in clearing misfolded proteins as well as a variety of cell material within exosomes and diffusible retromers (Figure 1). A knowledge of cell pathology produced by specific variants of VPS is relevant to describe the molecular mechanisms involved in abnormal cell-to-cell transmission. In keeping with this, it should be considered that VPS35 mutations represent an autosomal dominant genetic disorder. This suggests a pathological gain of function. Thus, one may hypothesize that an overactive exosome may increase retromers availability, thus contributing to the spreading of pathological cargoes along the CNS. If this hypothesis is correct one should expect that decreased retromer activity may exert a protective role. Unexpectedly, this is just the opposite of what has been recently indicated by Tang et al. (2015b), who demonstrated that, the loss of retromer activity leads to the loss of dopamine-containing neurons. Thus, it is likely that mutations leading to PD impair retromer activity, rather than providing an enhancement of retromer function. The dominant nature of these mutations imply that the activity of retromers needs to be highly preserved in order to keep cell homeostasis. Since the main function of retromers consists in transporting cell material from the plasma membrane to the trans Golgi network and back again, it is likely that even a slight disruption of this trafficking may impair cell survival. In fact, when assayed in the presence of an excess of alpha synuclein, in alpha synuclein transgenic mice, the concomitant over-expression of native VPS35 counteracts alpha-synuclein-dependent toxicity (Dhungel et al., 2015). In order to produce its beneficial effects VPS35 needs to be structurally intact since the up-regulation of mutant VPS35 worsens the neurotoxic effects produced by alpha synuclein (Dhungel et al., 2015). In light of these findings the effective activity of VPS appears to be critical to promote retromer function, thus providing a key step in the removal of toxic substrates (Wang et al., 2016; Figure 1). This is confirmed by Sowada et al. (2016) who showed protection from copper toxicity in yeast over-expressing VPS35, while copper and alpha-synuclein toxicity is enhanced in the same cells upon VPS35 dysfunction produced by VPS35 mutations. The natural function of VPS35 is bound to the PARK2 gene described as parkin (Kitada et al., 1998). In fact, despite multiple roles exerted by parkin as a E3 ubiquitin ligase (modulating both proteasome and autophagy activity), it seems that the most relevant effects are produced by its interaction with the endosomal compartment. Parkin was recently shown to regulate endosomal activity just based on its interaction with VPS35 (Song et al., 2016). These data strongly suggest that endocytic compartment is likely to be highly relevant in PD. This concept does not rule out previous findings showing both proteasome and autophagosome dysfunction. In fact all these compartments eventually merge to produce an ultimate organelle which fuses with lysosomes (Lenzi et al., 2016). The specific fate of endosomes is related to the chance that, despite routinely shuttling toward the trans Golgi network, this compartment may be delivered to the cell membrane to be released. This is effectively commented by Zhang and Schekman (2013) under the title “unconventional secretions, unconventional solutions.” Thus, we may consider that a dysfunction in the VPS complex shifts the routine trafficking from endosomes and trans Golgi network to the cell membrane to produce the release of toxic cargoes (Figure 1). In keeping with this hypothesis we may argue that a mutation in the VPS complex produces an abnormal cargoes release since the natural intracellular trafficking is no longer able to clean the cells from overwhelming retromers. This may explain why Song et al. (2016) described the occurrence of abnormal endosomes within parkin deficient cells where the endosomal cargoes were delivered to the extracellular space to be released. The emphasis which derives from recent findings on the pivotal role of the endosomal/retromer/exosome compartment in PD pathogenesis remains to be clearly balanced. For instance, recent studies demonstrate that a mutation of VPS35 alters the mitochondrial turnover (Tang et al., 2015a,b; Wang et al., 2016). These findings pose uncertain outcomes, which remain difficult to explain simply based on the current knowledge on the endosomal compartment. Understanding the significance of various mutations of VPS is key to dissect the site-specific relevance of each specific VPS isoform and it may help to understand why certain VPS mutations are associated with PD while others are linked to AD or may produce mixed disease phenotypes known as atypical PD.

## Author contributions

SG Coordinator of the sections about genetic and VPS genes. Participate in drafting. FB Pubmed research and state of the art about genetic of VPS26a,26b,29. RF Pubmed research and state of the art about genetic of VPS35 in PARK17. CB Pubmed research and state of the art about genetic of VPS26a,26b,29. AF Participate in critically revising the article for important intellectual content. SR Participate in drafting the article. Participate in critically revising the article for important intellectual content. GN Participate in critically revising the article for important intellectual content. FF Coordinator of the paper. Participate in drafting the article. Participate in critically revising the article for important intellectual content. Coordinator of the section “VPS and neurobiology of PD.”

### Conflict of interest statement

The authors declare that the research was conducted in the absence of any commercial or financial relationships that could be construed as a potential conflict of interest.
